# Developing and validating a machine learning model for predicting post-thrombolysis seizures in acute ischemic stroke

**DOI:** 10.1097/MD.0000000000047587

**Published:** 2026-02-06

**Authors:** Liangliang Jia, Yueqin Hu, Guilan Jin, Zhiyong Zhou

**Affiliations:** a Department of Pharmacy, Yichang Central People’s Hospital, Yichang, Hubei, China; b Institute of Pharmaceutical Preparation, China Three Gorges University, Yichang, Hubei, China; c Yichang Pharmaceutical Sociey, Yichang, Hubei, China; d Department of Pharmacy, College of Medicine and Health Sciences, China Three Gorges University, Yichang, Hubei, China.

**Keywords:** interpretability, machine learning, post-stroke seizures, prediction model, thrombolysis

## Abstract

Post-stroke seizures (PSS) manifests variably due to ischemic brain injury, yet its risk factors remain unclear. This study developed a machine learning (ML) model using clinical and laboratory data to predict PSS risk in acute ischemic stroke (AIS) patients post-thrombolysis, aiming to enhance risk assessment and clinical management. Retrospective analysis included 332 AIS patients treated between January 2020 and November 2024. Twenty-one variables (demographics, clinical parameters, lab biomarkers) were analyzed. Establish a diagnostic model with the occurrence of seizures after thrombolytic surgery as the classification variable. Missing data were handled via median/mean substitution, and class imbalance was corrected using synthetic minority oversampling technique. Feature selection combined expert consensus and Boruta algorithm. The dataset was split into training (70%) and testing (30%) cohorts. Seven ML models – logistic regression, Naïve Bayes, support vector machines, multilayer perceptron, AdaBoost, gradient boosting decision tree, and random forest (RF) – were evaluated using area under the curve (AUC), Brier score, accuracy, sensitivity, and specificity. Shapley Additive exPlanations (SHAP) analysis interpreted feature importance. PSS occurred in 39 patients (11.7%). Four predictors were identified: age, serum sodium, serum calcium, and fasting blood glucose. The RF model achieved optimal performance (AUC: 0.867, 95% CI: 0.793–0.930); accuracy: 0.803 (95% CI: 0.73–0.869); specificity: 0.810 (95% CI: 0.71–0.898), F1-score: 0.797 (95% CI: 0.711–0.87); positive predictive value: 0.797 (95% CI: 0.691–0.897), Kappa: 0.606 (95% CI: 0.441–0.739)). SHAP ranked fasting blood glucose as the strongest predictor, followed by serum sodium, serum calcium, and age. Lower electrolyte levels, elevated glucose, and younger age correlated with higher PSS risk. The model was deployed as a web-based clinical tool. The RF-based model effectively stratifies PSS risk in thrombolysis-treated AIS patients using accessible clinical variables. SHAP interpretability underscores fasting glucose, serum sodium/calcium, and age as pivotal predictors, offering actionable insights for prevention and personalized care. This tool may aid early intervention strategies to mitigate PSS burden.

## 1. Introduction

Cerebrovascular disease accounts for approximately 50% of new-onset epilepsy cases in adults. Clinical evidence indicates that 2 to 14% of ischemic stroke patients develop post-stroke seizures (PSS).^[1]^ Over the past few decades, researchers both domestically and internationally have been committed to exploring the risk factors for PSS.Clinical prediction models serve as quantitative tools for disease risk prediction and benefit assessment, and the development of high-performance prediction models has become a hot topic in current research. Researchers from Switzerland and Taiwan have each developed specialized predictive models for PSS following AIS according to clinical prediction model guidelines. In 2018, Galovic and others^[2]^ used 1200 ischemic stroke patients from Switzerland to construct the SeLECT risk scoring system, and verified the model’s good performance in external cohorts from 3 different countries (total validation cohort area under the curve (AUC) of 0.77). In the same year, Chiand others^[3]^ from Taiwan retrospectively collected demographic and clinical data of 87,068 ischemic stroke patients, and established the PSEiCARe score, which includes 7 clinical variables, aiming to predict PSS risk within one year after the onset of ischemic stroke (validation cohort AUC of 0.792). However, these prediction models are all based on regression algorithms and include relatively homogeneous populations, thus the accuracy and generalizability of the prediction models still need to be improved.

Therefore, this study retrospectively collected relevant medical data from patients with AIS at our hospital. Using machine learning (ML) methods, we constructed a predictive model for PSS occurring after thrombolysis treatment for AIS. The aim is to help identify high-risk individuals, develop preventive strategies, and ultimately assist in clinical decision-making.

## 2. Materials and methods

### 2.1. Participants

This study was approved by the Institutional Ethics Committee of Yichang Central People’s Hospital (Approval Date: April 25, 2025; Approval No: 2025-155-01). Due to the retrospective nature of the study, the requirement for written informed consent was waived by the committee. We conducted a retrospective cohort study of acute ischemic stroke (AIS) patients receiving intravenous thrombolysis at our hospital between January 2020 and November 2024 (N = 418). Diagnostic criteria: AIS was diagnosed according to the Chinese Guidelines for the Diagnosis and Treatment of Acute Ischaemic Stroke (2018): sudden onset of focal or global neurological deficits persisting for ≥24 hours, with intracranial hemorrhage excluded by CT or MRI and a corresponding responsible lesion identified; Post-stroke seizure (PSS): PSS was defined as a seizure occurring <7 days after stroke onset in patients without prior epilepsy or seizure history, after exclusion of other intracranial or systemic disorders, and with epileptiform discharges on EEG that topographically corresponded to the stroke lesion. Inclusion criteria: Diagnosis compliant with Chinese Guidelines for Acute Ischemic Stroke Management (2018); Intravenous alteplase administration within 4.5 hours of symptom onset; and Post-thrombolysis antiepileptic drug initiation for seizure management. Exclusion criteria: preexisting epilepsy or seizure disorders; history of intracranial hemorrhage, subarachnoid hemorrhage, or neurotrauma; intracranial neoplasms or CNS infections; history of autoimmune disease or blood disease; history of severe organ failure (heart, lung, liver or kidney); history of malignant tumors; incomplete clinical documentation; and in-hospital mortality. Finally, 332 patients were included in this study. This sample size met the minimum statistical requirement and supported the validity and robustness of theanalytical results.

### 2.2. Data collection and outcome measures

Thirty-one candidate predictors were evaluated, including lipid profiles (total cholesterol, LDL-C), coagulation markers (fibrinogen, D-dimer), metabolic parameters (fasting glucose, HbA1c, homocysteine, uric acid, serum creatinine, folate, lactate dehydrogenase),serum electrolytes (serum sodium concentration, serum calcium concentration), and comorbidities(hypertension, diabetes, pulmonary infection, coronary artery disease, and use of 5-hydroxytryptamine receptor inhibitor drugs) and demographic characteristics(gender, age). Head CT and MRI were performed in all patients, and infarction size was calculated according to the Pullicino formula. PSS was defined as epileptic seizures occurring within one week after a stroke.

### 2.3. Feature engineering

Establish a diagnostic model with the occurrence of seizures after thrombolytic surgery as the classification variable. Data preprocessing: Variables in which a single value accounted for more than 90% of the total were removed; and variables with a coefficient of variation >0.01 were removed. Outlier handling: For outliers identified based on quantiles, the capping/flooring method was adopted. Specifically, outliers below the lower bound were replaced with the lower bound value, while outliers above the upper bound were replaced with the upper bound value. Missing values were handled in 2 types: Variables with missing values exceeding 90% were deleted; continuous variables were imputed with the mean; categorical variables were imputed with multiple imputation. Class imbalance was mitigated through synthetic minority oversampling technique. Finally, 22 features were selected by integrating clinical expert consensus (Delphi method) and the Boruta wrapper algorithm.

### 2.4. Model development

Seven ML algorithms were implemented via scikit-learn (v1.6.1): logistic regression, Naïve Bayes, support vector machine, multilayer perceptron, AdaBoost, gradient boosting decision tree, random forest (RF). Initially, the default hyperparameters of each ML model were adopted. To effectively mitigate model overfitting, systematic tuning was subsequently performed on the hyperparameters of the 7 ML models via Grid Search combined with a 5-fold cross-validation strategy.

### 2.5. Model evaluation and validation

The performance of the 7 models were comprehensively evaluated based on internal validation. The following 10 indicators were used to evaluate the model performance: accuracy, precision, recall, F1-score, sensitivity, specificity, positive predictive value (PPV), negative predictive value, and Kappa value. The higher these values are, the better the model performance. Conversely, for the Brier score, the closer the score is to 0, the higher the accuracy of the prediction model. Generally, a Brier score < 0.15 indicates that the model has good consistency. In addition, the area under the receiver operating characteristic curve (AUC) was used as the main evaluation indicator to assess the discrimination performance of the 7 prediction models. The higher the AUC value, the better the classification performance of the model. A calibration curve was introduced to calculate the predicted probability of a given model.

### 2.6. SHAP visualization for interpreting ML models

Shapley Additive exPlanations (SHAP) is a game - theoretic method used to interpret the output results of ML models. SHAP allows for the assignment of values to each input feature variable in the prediction model, that is, SHAP values. SHAP values can be interpreted as the importance of feature variables, reflecting the impact of feature variables on the prediction outcome. The higher the positive or negative SHAP value, the more important a certain feature variable is. A positive value indicates that the feature variable will increase the output of the model, a negative value indicates that the output of the model will decrease, and a SHAP value close to 0 indicates that the variable has a low impact on the prediction result. A SHAP summary plot was constructed based on SHAP values to rank the importance of risk factors. In the SHAP summary plot, the length of the horizontal axis where each variable is located represents the contribution of the variable to the prediction result, and the color of the points represents the size or category of the variable. A SHAP dependence plot was constructed to help understand the behavior of the model within different ranges of feature values. If the SHAP value monotonically increases with the increase of a certain feature value, it indicates that the model believes that this feature has a positive correlation with the prediction result; if the curve fluctuates or has an inflection point, it may mean that the decision - making logic of the model is different in different intervals of feature values.

### 2.7. Statistical analysis

In this study, a comparative analysis was conducted on the characteristics of patients included in the training set and the validation set. The Shapiro–Wilk test was used to assess the normality of all continuous variables. Variables following a normal distribution are expressed as “mean ± standard deviation,” with intergroup comparisons conducted using the independent samples *t*-test; variables not following a normal distribution are presented as “median (interquartile range, IQR),” and intergroup comparisons are performed using the Wilcoxon rank-sum test. Categorical variables were presented as count (percentage), and intergroup comparisons were performed using the χ^2^ test.

Patients were randomly allocated to the training set and the validation set at a ratio of 7:3. Seven ML models were trained in the training set, and the discriminative performance of the models was evaluated by calculating the median and 95% confidence interval of the area under the receiver operating characteristic curve (AUROC). Meanwhile, accuracy, sensitivity, specificity, negative predictive value, and PPV were also calculated. All statistical analyses in this study were performed using SPSS (version 25.0) or Python (version 3.12.4).

### 2.8. The presentation and application of the model

The model is deployed as a web application on a website for clinical use. The web address is as follows: http://clinicaldata.free.nf/.

## 3. Results

### 3.1. Cohort characteristics

**Figure 1.**
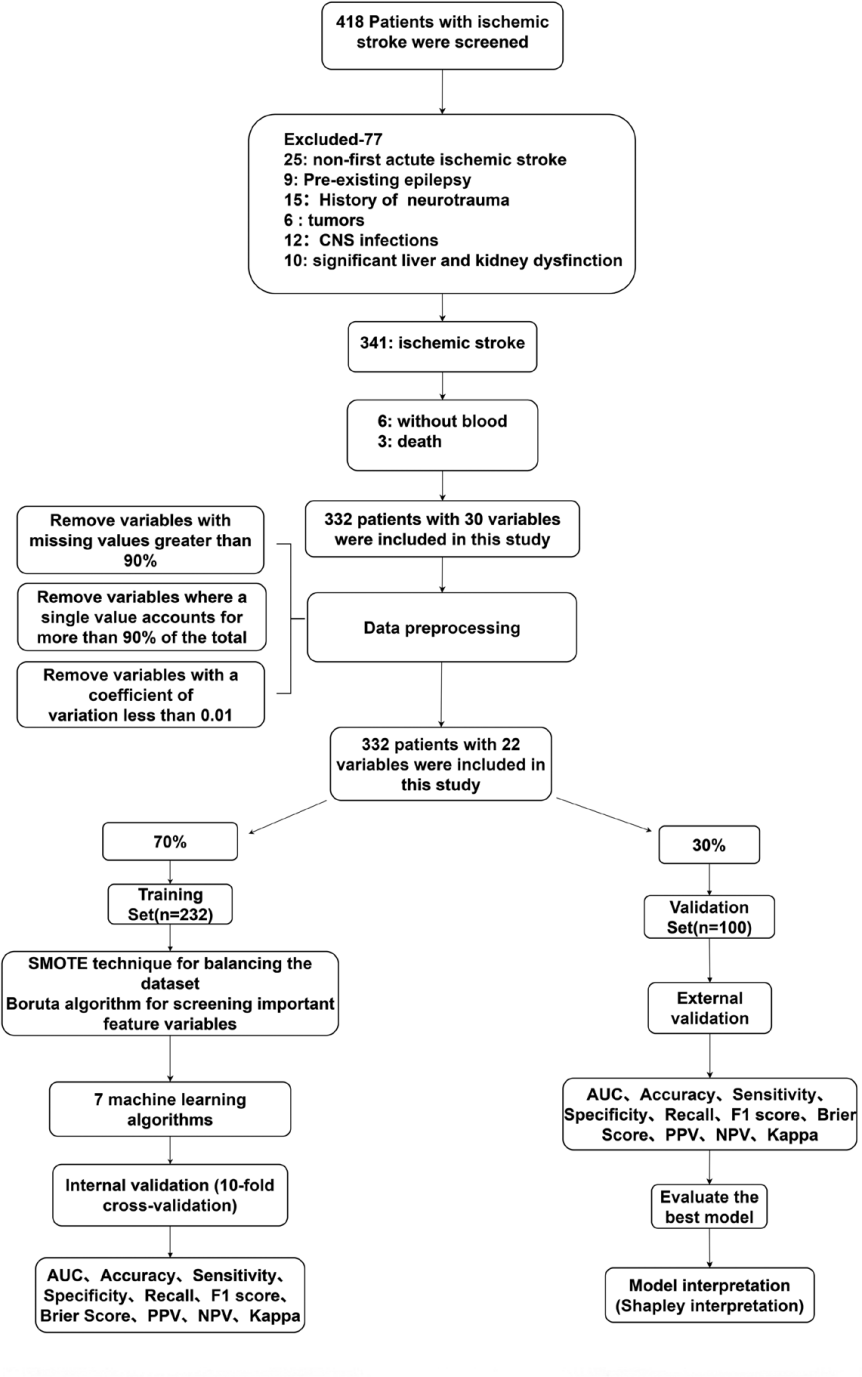
A study flow diagram. AUC = area under the curve, CNS = central nervous system, NPV = negative predictive value, PPV = positive predictive value, SMOTE = synthetic minority oversampling technique.

The study cohort comprised 332 consecutive AIS patients undergoing intravenous thrombolysis (60.2% males; mean age 70.0 [interquartile range, IQR: 62.0–77.0] years). Post-thrombolysis seizures developed in 39 patients (11.7%). Baseline characteristics stratified by seizures status are detailed in Table 1. The flow chart of our study is shown in Figure 1.

**Table 1:** Baseline Characteristics of the research cohort.

Feature	Total	No PSS	PSS	%NaN values (%)	*P*-value	Statistical test
	n = 332	n = 293	n = 39			
Gender (Male)	200 (60.2%)	173 (59.0%)	27 (69.2%)	1.81	.37	Chi-square tests
Age (yr)	70.0 (62.0, 77.0)	71.0 (57.0, 75.0)	70.0 (62.0, 78.0)	1.81	.23	Wilcoxon rank-sum tests
Hospitalization duration (d)	12.0 (8.0, 16.0)	15.0 (10.0, 25.5)	12.0 (8.0, 16.0)	1.81	**<.001**	Wilcoxon rank-sum tests
Hypertension	256 (77.1%)	233 (79.5%)	23 (59.0%)	0	**<.001**	Chi-square tests
Diabetes mellitus	106 (31.9%)	96 (32.8%)	10 (25.6%)	0	**.01**	Chi-square tests
Coronary artery disease	44 (13.3%)	39 (13.3%)	5 (12.8%)	0	.48	Chi-square tests
Pulmonary infection	44 (13.3%)	36 (12.3%)	8 (20.5%)	0	>.05 (*P* = .999)	Chi-square tests
Use of 5-HT	10 (3.0%)	8 (2.7%)	2 (5.1%)	0	.24	Chi-square tests
TC (mmol/L)	4.06 (3.48, 4.87)	4.27 ± 1.24	4.11 (3.48, 4.86)	0	.97	Wilcoxon rank-sum tests
LDL-C (mmol/L)	2.53 (1.91, 3.24)	2.45 (2.22, 3.44)	2.6 ± 0.88	31.02	.55	Wilcoxon rank-sum tests
Fib (g/L)	3.05 (2.62, 3.5)	3.21 (2.81, 3.79)	3.02 (2.57, 3.48)	46.69	.07	Wilcoxon rank-sum tests
D-Dimer (ng/mL)	602.0 (4.0, 1776.5)	723.0 (7.94, 1633.5)	596.0 (3.64, 1862.0)	20.78	.56	Wilcoxon rank-sum tests
Hcy (µmol/L)	13.15 (10.32, 16.87)	13.7 ± 5.06	13.07 (10.35, 16.62)	20.48	.97	Wilcoxon rank-sum tests
FBG (mmol/L)	5.45 (4.77, 6.74)	6.17 ± 1.51	5.41 (4.76, 6.7)	38.25	.27	Wilcoxon rank-sum tests
Folate (ng/mL)	6.09 (4.05, 9.24)	6.0 (4.1, 7.8)	6.15 (4.05, 9.69)	36.75	.56	Wilcoxon rank-sum tests
HbA1c (%)	5.8 (5.4, 6.25)	5.73 ± 0.69	5.8 (5.4, 6.3)	37.65	.16	Wilcoxon rank-sum tests
SUA (µmol/L)	324.31 ± 94.14	310.01 ± 71.67	326.57 ± 97.17	30.42	.33	*t*-tests
SCr (µmol/L)	77.5 (63.0, 91.77)	76.5 (69.5, 90.55)	77.5 (61.92, 91.75)	22.89	.47	Wilcoxon rank, sum tests
Na^+^ (mmol/L)	140.4 (139.0, 142.09)	140.66 ± 3.01	140.48 (139.0, 142.05)	22.59	.94	Wilcoxon rank-sum tests
Ca^2+^ (mmol/L)	2.21 (2.13, 2.29)	2.18 ± 0.12	2.21 (2.14, 2.3)	22.29	.05	Wilcoxon rank-sum tests
LDH (U/L)	192.5 (167.12, 228.5)	205.0 (182.0, 237.0)	191.5 (166.0, 226.0)	22.59	.12	Wilcoxon rank-sum tests
NIHSS scores	3 (2, 7)	3 (2, 7)	4 (2, 7.5)	38.25	.63	Chi-square tests

Variables following a normal distribution are expressed as “mean ± standard deviation (SD),” with intergroup comparisons conducted using the independent samples *t*-test; Variables not following a normal distribution are presented as “median (interquartile range, IQR),” and intergroup comparisons are performed using the Wilcoxon rank-sum test. Categorical variables were presented as count (percentage), and intergroup comparisons were performed using the χ^2^ test.

5-HT = 5-hydroxytryptamine, Ca^2+^ = serum calcium concentration, D-Dimer = D-dimer, FBG = fasting blood glucose, Fib = fibrinogen, Folate = folate, HbA1c = glycosylated hemoglobin, Hcy = homocystein, LDH = lactate dehydrogenase, LDL-C = low-density lipoprotein cholesterol, Na^+^ = serum sodium concentration, NIHSS = National Institutes of Health Stroke Scale, PSS = post-stroke seizures, SCr = serum creatinine, SUA = serum uric acid, TC = total cholesterol.

### 3.2. Model development

Feature selection through Boruta algorithm and clinical consensus identified 4 predictive variables: age, serum sodium, serum calcium, and fasting glucose. Seven ML classifiers were subsequently trained using these predictors.

**Figure 2.**
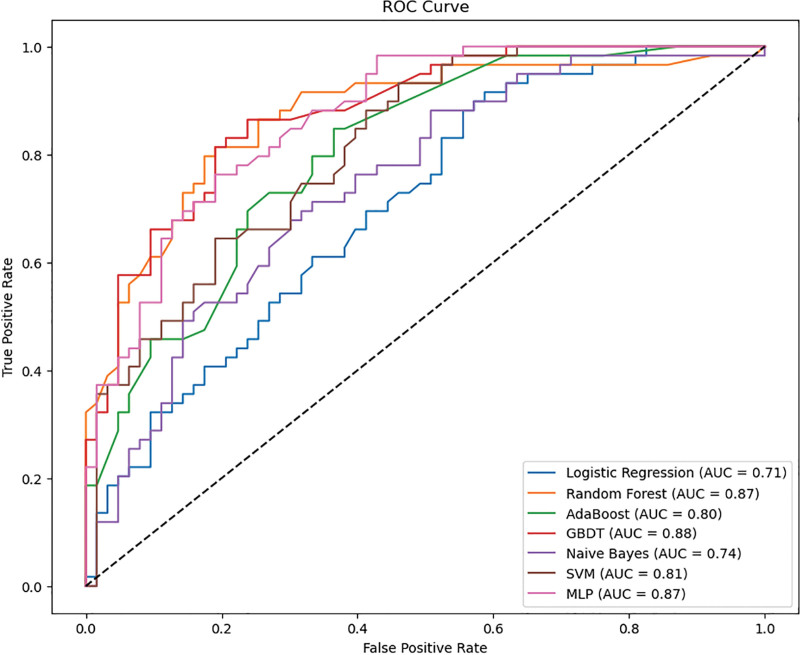
ROC curves of models constructed by 7 algorithms in the test set. AUC = area under the curve, GBDT = gradient boosting decision tree, MLP = multilayer perceptron, ROC = receiver operating characteristic, SVM = support vector machines.

**Figure 3.**
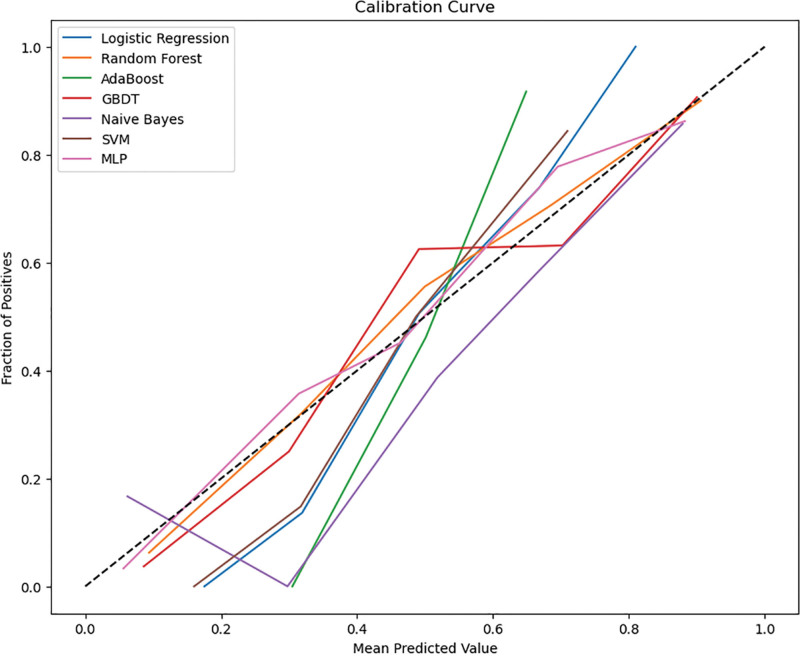
Calibration curves of 7 machine learning models on the test set. GBDT = gradient boosting decision tree, MLP =multilayer perceptron, SVM = support vector machines.

A prediction model was constructed based on the selected 4 feature variables. All models had good stability, and there was no obvious overfitting or underfitting in each model. In the training set, the performance indicators of the RF model were better than those of other models, as shown in Table 2. In the test set, 5 performance indicators of the RF model (accuracy: 0.803 (95% CI: 0.73–0.869), specificity: 0.810 (95% CI: 0.71–0.898), F1-score: 0.797 (95% CI: 0.711–0.87), PPV: 0.797 (95% CI: 0.691–0.897), Kappa: 0.606 (95% CI: 0.441–0.739)) were better than those of other models, and its average AUC value on the test set (AUC = 0.867 (95% CI: 0.793–0.930)) was the highest. This reflects that the RF model performs well in avoiding overfitting or underfitting (Fig. 2). A calibration curve was introduced to evaluate the consistency between the predicted probability and the actual occurrence probability of the model. The results showed that the calibration curve of the RF model on the test set had higher prediction accuracy (Fig. 3).

**Table 2:** Performance evaluation indicators of models constructed by 7 algorithms.

Algorithm	AUC (95% CI)	Accuracy (95% CI)	Recall (95% CI)	Sensitivity (95% CI)	Specificity (95% CI)
Training set					
Logistic regression	0.642 (0.58–0.702)	0.606 (0.549–0.662)	0.639 (0.563–0.718)	0.639 (0.557–0.714)	0.571 (0.489–0.657)
Random forest	0.998 (0.995–1.0)	0.986 (0.972–0.996)	0.979 (0.956–1.0)	0.979 (0.95–1.0)	0.993 (0.976–1.0)
AdaBoost	0.859 (0.813–0.901)	0.813 (0.764–0.863)	0.792 (0.721–0.854)	0.792 (0.728–0.852)	0.836 (0.77–0.895)
GBDT	0.988 (0.98–0.996)	0.954 (0.93–0.975)	0.938 (0.893–0.975)	0.938 (0.896–0.972)	0.971 (0.94–0.993)
Naive Bayes	0.661 (0.598–0.722)	0.627 (0.574–0.68)	0.882 (0.826–0.931)	0.882 (0.83–0.932)	0.364 (0.289–0.444)
SVM	0.835 (0.786–0.876)	0.722 (0.669–0.778)	0.743 (0.673–0.811)	0.743 (0.672–0.813)	0.7 (0.619–0.774)
MLP	0.952 (0.928–0.972)	0.877 (0.841–0.912)	0.861 (0.804–0.918)	0.861 (0.801–0.912)	0.893 (0.838–0.941)
Test set					
Logistic regression	0.705 (0.605–0.8)	0.631 (0.541–0.713)	0.61 (0.482–0.731)	0.61 (0.474–0.734)	0.651 (0.528–0.758)
Random forest	0.867 (0.793–0.93)	0.803 (0.73–0.869)	0.797 (0.687–0.895)	0.797 (0.696–0.897)	0.81 (0.71–0.898)
AdaBoost	0.804 (0.727–0.878)	0.73 (0.648–0.803)	0.729 (0.615–0.836)	0.729 (0.611–0.839)	0.73 (0.619–0.836)
GBDT	0.867 (0.816–0.932)	0.803 (0.738–0.869)	0.797 (0.697–0.898)	0.797 (0.696–0.895)	0.81 (0.71–0.903)
Naive Bayes	0.745 (0.654–0.824)	0.639 (0.549–0.73)	0.932 (0.867–0.984)	0.932 (0.857–0.984)	0.365 (0.25–0.485)
SVM	0.814 (0.733–0.879)	0.697 (0.607–0.771)	0.661 (0.528–0.778)	0.661 (0.55–0.781)	0.73 (0.617–0.841)
MLP	0.856 (0.79–0.915)	0.746 (0.664–0.82)	0.695 (0.582–0.806)	0.695 (0.576–0.8)	0.794 (0.692–0.883)
**Algorithm**	**Brier score (95% CI**)	**F1 score (95% CI**)	**PPV (95% CI**)	**NPV (95% CI**)	**Kappa (95% CI**)
Training set					
Logistic regression	0.234 (0.221–0.247)	0.622 (0.554–0.685)	0.605 (0.521–0.684)	0.606 (0.521–0.686)	0.21 (0.092–0.327)
Random forest	0.028 (0.022–0.036)	0.986 (0.97–0.997)	0.993 (0.978–1.0)	0.979 (0.952–1.0)	0.972 (0.943–0.993)
AdaBoost	0.21 (0.203–0.216)	0.811 (0.763–0.857)	0.832 (0.771–0.892)	0.796 (0.733–0.859)	0.627 (0.528–0.712)
GBDT	0.052 (0.041–0.064)	0.954 (0.927–0.978)	0.971 (0.941–0.993)	0.938 (0.895–0.973)	0.908 (0.858–0.958)
Naive Bayes	0.23 (0.21–0.252)	0.706 (0.653–0.76)	0.588 (0.516–0.651)	0.75 (0.648–0.851)	0.248 (0.148–0.34)
SVM	0.172 (0.156–0.19)	0.73 (0.669–0.781)	0.718 (0.651–0.791)	0.726 (0.651–0.799)	0.443 (0.339–0.544)
MLP	0.098 (0.083–0.116)	0.876 (0.832–0.913)	0.892 (0.836–0.939)	0.862 (0.808–0.914)	0.754 (0.668–0.831)
Test set					
Logistic regression	0.219 (0.201–0.237)	0.615 (0.513–0.715)	0.621 (0.492–0.737)	0.641 (0.516–0.759)	0.261 (0.098–0.426)
Random forest	0.148 (0.113–0.183)	0.797 (0.711–0.87)	0.797 (0.691–0.897)	0.81 (0.714–0.903)	0.606 (0.441–0.739)
AdaBoost	0.217 (0.207–0.227)	0.723 (0.619–0.805)	0.717 (0.603–0.827)	0.742 (0.627–0.844)	0.459 (0.285–0.622)
GBDT	0.142 (0.112–0.174)	0.797 (0.714–0.873)	0.797 (0.692–0.902)	0.81 (0.708–0.893)	0.606 (0.449–0.737)
Naive Bayes	0.222 (0.192–0.254)	0.714 (0.628–0.786)	0.579 (0.474–0.677)	0.852 (0.711–0.967)	0.292 (0.155–0.418)
SVM	0.182 (0.16–0.205)	0.678 (0.577–0.769)	0.696 (0.583–0.815)	0.697 (0.581–0.803)	0.392 (0.229–0.541)
MLP	0.151 (0.119–0.182)	0.726 (0.621–0.814)	0.759 (0.64–0.873)	0.735 (0.623–0.836)	0.49 (0.344–0.639)

AdaBoost = adaptive boosting, AUC = area under the receiver operating characteristic curves, CI = confidence interval, GBDT = gradient boosting decision tree, MLP = multilayer perceptron, NPV = negative predictive value, PPV = positive predictive value, SVM = support vector machine.

### 3.3. Interpretability analysis of the prediction model for seizures after stroke thrombolysis

According to the verification results of the PSS prediction model, the RF algorithm model with the relatively best comprehensive prediction performance was finally selected as the object of interpretability analysis of the ML model. The SHAP tool was used for post-hoc interpretability analysis.

#### 3.3.1. Feature importance of the model

**Figure 4.**
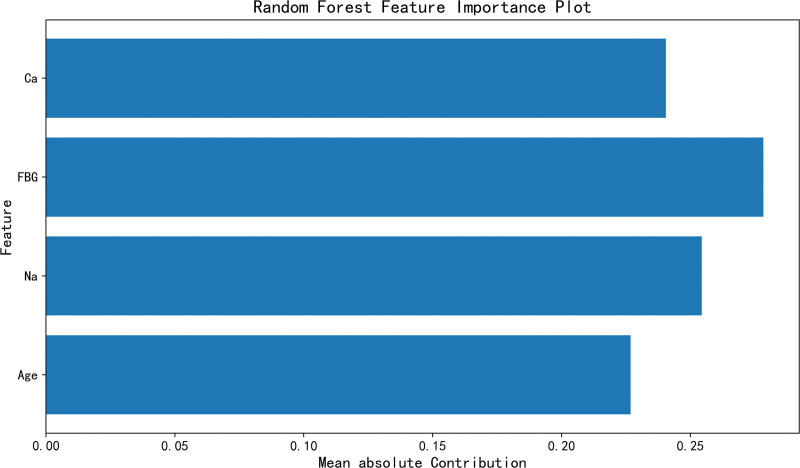
Random forest feature importance plot. Ca = serum calcium, FBG = fasting blood glucose, Na = serum sodium.

The SHAP tool ranked the importance of the 4 features included in the RF model by calculating the Shapley values, and simultaneously drew a feature importance plot and a dependence plot for visual presentation. The importance of the 4 features composing the model was ranked as fasting blood glucose, serum sodium concentration, serum calcium concentration, and age from the most important to the least. The more forward the ranking, the more important the feature is and the greater its contribution to the model (Fig. 4).

#### 3.3.2. Interpretive analysis of the role of features in the model

**Figure 5.**
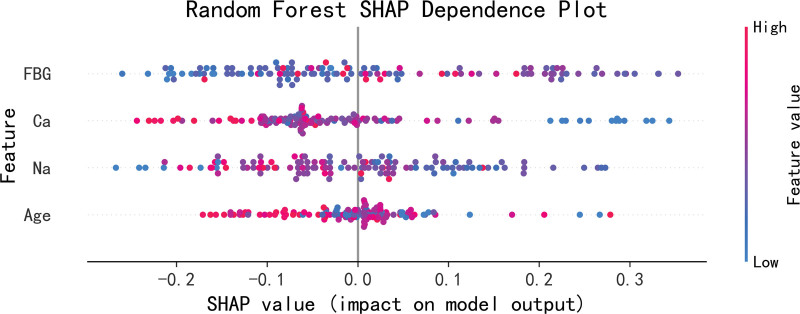
SHAP summary plot of the random forest model. Ca = serum calcium, FBG = fasting blood glucose, Na = serum sodium, SHAP = Shapley Additive exPlanations.

**Figure 6.**
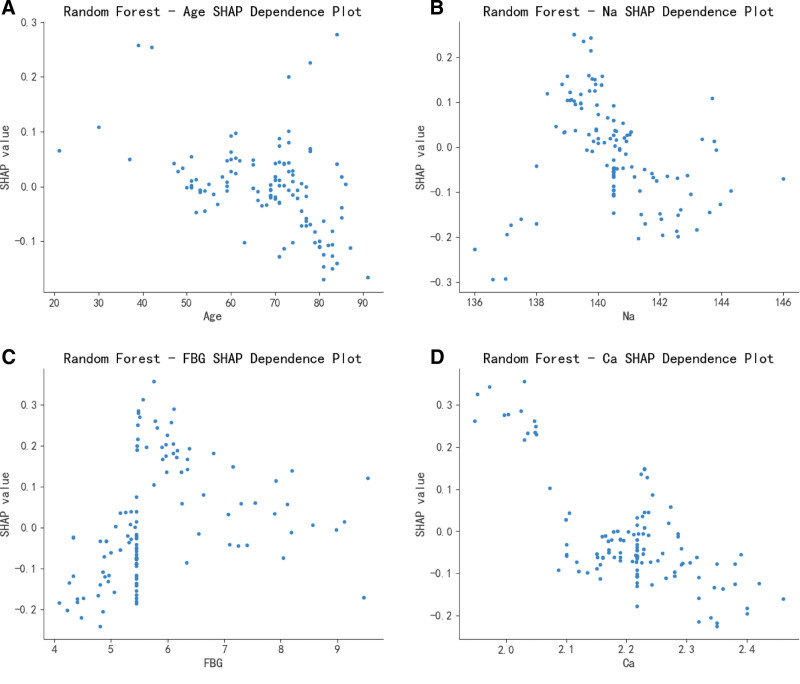
SHAP dependence plot of the random forest model. (A) The impact of the age factor on SHAP values in the random forest model. (B) The impact of the serum sodium concentration factor on SHAP values in the random forest model. (C) The impact of the fasting blood glucose factor on SHAP values in the random forest model. (D) The impact ofthe serum calcium concentration factor on SHAP values in the random forest model. Ca = serum calcium, FBG = fasting blood glucose, Na = serum sodium, SHAP = Shapley Additive exPlanations.

The SHAP value explains how features affect the prediction results of the model by reflecting the influence (Shapley value) of different features of each sample. As shown in Figure 4, in the SHAP value distribution plot, the ordinate represents the features, and the abscissa represents the Shapley values. When the Shapley value on the right side of the mid-line is positive, it indicates that the feature tends to predict a positive result, while when the Shapley value on the left side of the mid-line is negative, it means that the feature makes the model more inclined to a negative result. Each point in the figure represents a sample. The more discrete the points are, the more significant the influence of the feature; conversely, if the points are distributed on the mid-line, it indicates that the feature has a smaller impact on the outcome event. In addition, each sample point is marked in red or blue. Red indicates a higher feature value, and blue indicates a lower feature value. According to the drawn SHAP summary plot (Fig. 5) and dependence plot (Fig. 6), it was shown that a decrease in serum calcium and sodium levels increased the risk of seizures, an increase in blood glucose levels had a certain impact on the occurrence of seizures, and seizures tended to occur in young patients. All analyses were performed using Python, version 3.12.4.

## 4. Discussion

Post-ischemic stroke seizures (PSS) was caused by ischemic stroke damage in different regions of the brain, with diverse clinical manifestations. According to the time interval between stroke onset and the first occurrence of seizures, post - stroke seizures can be classified as early-onset seizure (ES) if epilepsy occurs within 1 week after stroke and late-onset seizure if it occurs more than 1 week after stroke.^[4]^ Some studies have reported that the incidence rate of ES is 2 to 33%, and that of late-onset seizure is 3 to 67%.^[5]^ In this study, 332 patients were included, and 39 patients had ESs during hospitalization after thrombolysis for AIS, with an incidence rate of 11.7%, which is basically consistent with the reported data.

Currently, the influencing factors for the onset of PSS are not clear. Some basic research suggests that the pathological mechanisms of early-onset SE include electrolyte imbalance, acid-base disorders, disruption of the phospholipid bilayer, increased release of the excitatory neurotransmitter glutamate, and oxidative stress and mitochondrial dysfunction.^[6,7]^ There is still some controversy regarding the risk factors for seizures. Existing studies believe that factors such as the patient’s age of onset, large-artery atherosclerotic stroke, hemorrhagic transformation, stroke complications, use of stroke prevention drugs, stroke severity, and cortical involvement^[1,8–11]^ may be related to early-onset SE.

In the past decade or so, researchers have attempted to develop clinical prediction tools to accurately identify high-risk patients with PSS. Previous studies have used classic regression algorithms in traditional statistical analysis methods to construct prediction models for post-ischemic stroke seizures. Although classic regression algorithms have been widely used in disease prediction, due to the requirements that feature variables need to meet different prerequisite assumptions (linearity, independence, normality, etc) and the limitations of hypothesis testing, the expression ability and application scope of the models are restricted, and the accuracy still needs to be improved.^[12]^

Machine learning, as one of the core technologies in the field of artificial intelligence, has the advantage of being able to handle complex non-linear relationships between data compared with classic regression algorithms, and it also has good stability in the prediction process. Currently, ML technology is being increasingly used in the medical field to solve various complex problems. However, the application of ML technology in the field of seizures is still in the exploratory stage. Especially for ESs after thrombolysis for ischemic stroke, no researchers at home and abroad have yet attempted to construct an effective prediction model using ML algorithms.

Therefore, we have developed a predictive model using ML methods to forecast the occurrence of seizures in patients with AIS after thrombolysis. The AUC value of the RF model constructed in this study (AUC = 0.867) was significantly higher than that of the models established by Galovi^[2]^ (AUC = 0.77) and Chi^[3]^ (AUC = 0.792) based on regression algorithms. This indicates that the model in this study has superior performance and can more accurately predict the occurrence of seizures. By expanding the usage scenarios of the predictive model through a visual web application, interventions targeting the corresponding risk factors can be made to reduce the likelihood of seizures in patients.

In this study, based on expert consultation suggestions and the Boruta algorithm, 21 variables including patient demographic characteristics, clinical data, and laboratory test index information were screened. Finally, 4 feature variables, including age, serum calcium concentration, serum sodium concentration, and fasting blood glucose, were selected for constructing the model. After feature selection, 7 classic ML algorithms were used to construct the PSS prediction model. Subsequently, in the test set and validation set cohort datasets, the prediction performance of the models was evaluated from 3 aspects: discrimination, calibration, and clinical utility, and the best model for predicting PSE was selected. Through comprehensive analysis of the above 3 aspects, the model constructed by the RF algorithm showed more excellent prediction performance than other ML models in terms of having the best discrimination (AUC), ideal calibration effect (Brier score), and good clinical utility (sensitivity, specificity).

This study used the SHAP tool for interpretability analysis of the model. The results showed that among the 4 features composing the model, the importance was ranked as fasting blood glucose, serum sodium concentration, serum calcium concentration, and age from the most important to the least.

Fasting blood glucose is one of the important feature variables selected in this study for constructing the ML model. Research has found that hyperglycemia is a high-risk factor for ischemic stroke.^[1]^ Hyperglycemia can cause pathological changes in cerebral blood vessels, aggravating the degree of brain tissue damage; it can increase anaerobic glycolysis in cells, leading to an increase and accumulation of lactic acid, aggravating neuronal damage^[13]^; and hyperglycemia significantly upregulates TLR4-mediated neuroinflammation, thereby increasing the risk of cerebral and neuronal damage as well as delay in neurofunctional recovery.^[14,15]^ All of these factors can not only exacerbate cerebral ischemia-reperfusion injury but also significantly increase the incidence of PSS. In this study, the results of the SHAP summary plot and dependence plot showed that an increase in blood glucose levels had a certain impact on the occurrence of seizures.

The electrolyte levels of patients played an important role in the prediction of PSS. Basic research also suggests that low calcium levels may lead to abnormal discharges and cause EOS through excessive Ca^2+^ influx, resulting in excessive depolarization of neurons. Some studies have found in animal models that the extracellular Ca^2+^ level decreases before epileptic seizures, indicating that excessive Ca^2+^ influx is closely related to epileptic seizures.^[16]^ Excessive Ca^2+^ influx occurs through voltage-dependent calcium channels and receptor-operated calcium channels. There are also studies indicating that the body is more prone to hyponatremia and hypocalcemia after a stroke. This can exacerbate brain edema and brain damage, lower the seizure threshold, and thus induce epileptic seizures. When stroke patients are complicated with hyponatremia, hyponatremia can cause damage to the nervous system and trigger seizures.^[17,18]^ This study suggests that decreased serum calcium and sodium levels increase the risk of epileptic seizures.

In this study, the patient’s age also played an important role in the prediction of PSS. Some studies have suggested that age is one of the risk factors for the occurrence of PSS, and advanced age is independently associated with a reduced risk of PSS.^[19]^ A retrospective study of 1880 patients hospitalized for their first stroke found that age was a relevant factor for PSS.^[20]^ An epidemiological and correlation analysis study of 3310 patients in a stroke registry in South London found that youth was associated with PSS.^[21]^ Other studies have found that the incidence rate and seizure frequency of PSS in elderly stroke patients aged < 74 years were significantly higher than those in patients aged ≥ 75 years.^[22]^ Some studies believe that with increasing age, the shrinkage of the cerebral cortex and degenerative changes in the elderly can lead to a decrease in cortical excitability, thus reducing the risk and severity of PSS.^[23]^ The average age of the patients included in this study was 70 years old, and the research results also showed that seizures tended to occur in young patients.

## 5. Limitations

This study has some limitations: Using a retrospective cohort to construct the model may introduce selection bias. In contrast, a prospective dataset is more suitable for improving the predictive ability of the model; All clinical data in this study were derived from inspection reports, so information bias was inevitable; Since this study was a single-center retrospective study, there were certain limitations in sample representativeness and sample size. The study of risk factors for PSS and the improvement of prediction models require a larger sample size and more comprehensive clinical data. Moreover, the lack of external validation means that the research results need to be verified in other institutions to ensure their reliability.

## 6. Conclusions

In conclusion, the present study demonstrates that the RF model exhibits satisfactory predictive performance for post-stroke epilepsy, with 3 key risk factors identified: blood glucose levels during thrombolysis, electrolyte status, and age (patients under 75 years old). The identification of these factors highlights the potential clinical relevance of targeted monitoring and risk stratification in stroke patients. Specifically, the findings suggest that attention should be paid to blood glucose regulation, electrolyte balance maintenance, and enhanced clinical surveillance for younger patients (under 75 years old) in clinical practice, as these aspects may be closely associated with the risk of post-stroke epilepsy.

Given that the present study is a predictive modeling study, the potential effectiveness of specific interventions targeting these identified factors remains to be validated through empirical research. Therefore, the research team plans to conduct subsequent work and prospective studies in collaboration with multiple centers in the future. After identifying patients at potential risk of PSS, proactive interventions will be implemented, followed by long-term follow-up to document intervention outcomes. This will further evaluate and refine the predictive model, with the aim of facilitating the clinical diagnosis and management of PSS.

Overall, this study provides a satisfactory predictive tool for post-stroke epilepsy and identifies actionable risk factors that may inform future clinical research and hypothesis generation. Further prospective studies are warranted to verify the clinical utility of interventions targeting the identified risk factors and to establish evidence-based strategies for post-stroke epilepsy prevention.

## Acknowledgments

This project was supported by the Second Batch of Comprehensive Evaluation Projects for Clinical Drugs of the Health Commission of Hubei Province in terms of technical guidance. We also thank all the participants and collaborators who contributed to this study.

## Author contributions

**Data curation:** Liangliang Jia, Yueqin Hu, Guilan Jin, Zhiyong Zhou.

**Formal analysis:** Liangliang Jia, Zhiyong Zhou.

**Investigation:** Liangliang Jia, Zhiyong Zhou.

**Methodology:** Liangliang Jia, Yueqin Hu, Zhiyong Zhou.

**Software:** Liangliang Jia, Yueqin Hu, Guilan Jin.

**Writing – original draft:** Liangliang Jia, Yueqin Hu.

**Writing – review & editing:** Liangliang Jia, Yueqin Hu.

## References

[1] Pitkänen A Roivainen R Lukasiuk K. Development of epilepsy after ischaemic stroke. Lancet Neurol. 2016;15:185–97. 26597090 10.1016/S1474-4422(15)00248-3

[2] Galovic M Döhler N Erdélyi-Canavésse B. Prediction of late seizures after ischaemic stroke with a novel prognostic model (the SeLECT score): a multivariable prediction model development and validation study. Lancet Neurol. 2018;17:143–52. 29413315 10.1016/S1474-4422(17)30404-0

[3] Chi NF Kuan YC Huang YH. Development and validation of risk score to estimate 1-year late poststroke epilepsy risk in ischemic stroke patients. Clin Epidemiol. 2018;10:1001–11. 30174459 10.2147/CLEP.S168169 PMC6110266

[4] Beghi E Carpio A Forsgren L. Recommendation for a definition of acute symptomatic seizure. Epilepsia. 2010;51:671–5. 19732133 10.1111/j.1528-1167.2009.02285.x

[5] Chen TC Chen YY Cheng PY Lai C-H. The incidence rate of post-stroke seizures: a 5-year follow-up study in Taiwan. Epilepsy Res. 2012;102:188–94. 22749919 10.1016/j.eplepsyres.2012.06.003

[6] Altman K Shavit-Stein E Maggio N. Post stroke seizures and epilepsy: from proteases to maladaptive plasticity. Front Cell Neurosci. 2019;13:397. 31607864 10.3389/fncel.2019.00397 PMC6755337

[7] Rowley S Patel M. Mitochondrial involvement and oxidative stress in temporal lobe epilepsy. Free Radic Biol Med. 2013;62:121–31. 23411150 10.1016/j.freeradbiomed.2013.02.002 PMC4043127

[8] Ferlazzo E Gasparini S Beghi E; Epilepsy Study Group of the Italian Neurological Society. Epilepsy in cerebrovascular diseases: review of experimental and clinical data with meta-analysis of risk factors. Epilepsia. 2016;57:1205–14. 27381481 10.1111/epi.13448

[9] Bladin CF Alexandrov AV Bellavance A. Seizures after stroke: a prospective multicenter study. Arch Neurol. 2000;57:1617–22. 11074794 10.1001/archneur.57.11.1617

[10] Zhao L Li J Kälviäinen R Jolkkonen J Zhao C. Impact of drug treatment and drug interactions in post-stroke seizures. Pharmacol Ther. 2022;233:108030. 34742778 10.1016/j.pharmthera.2021.108030

[11] Gasparini S Ascoli M Brigo F. Younger age at stroke onset but not thrombolytic treatment predicts poststroke epilepsy: an updated meta-analysis. Epilepsy Behav. 2020;104(Pt B):106540. 31677999 10.1016/j.yebeh.2019.106540

[12] Christodoulou E Ma J Collins GS Steyerberg EW Verbakel JY Van Calster B. A systematic review shows no performance benefit of machine learning over logistic regression for clinical prediction models. J Clin Epidemiol. 2019;110:12–22. 30763612 10.1016/j.jclinepi.2019.02.004

[13] Yong M Kaste M. Dynamic of hyperglycemia as a predictor of stroke outcome in the ECASS-II trial. Stroke. 2008;39:2749–55. 18703813 10.1161/STROKEAHA.108.514307

[14] Oo TT. Ischemic stroke and diabetes: a TLR4-mediated neuroinflammatory perspective. J Mol Med (Berl). 2024;102:709–17. 38538987 10.1007/s00109-024-02441-9

[15] Vezzani A Bartfai T Bianchi M Rossetti C French J. Therapeutic potential of new antiinflammatory drugs. Epilepsia. 2011;52(Suppl 8):67–9. 21967368 10.1111/j.1528-1167.2011.03242.x

[16] Pumain R Kurcewicz I Louvel J. Fast extracellular calcium transients: involvement in epileptic processes. Science. 1983;222:177–9. 6623068 10.1126/science.6623068

[17] Hui-Min L Ming-Long L. Research progress in diagnosis and treatment of hyponatraemia. Chin J Mult Organ Dis Elderly. 2018;17:233–6.

[18] Roivainen R Haapaniemi E Putaala J Kaste M Tatlisumak T Young adult ischaemic stroke related acute symptomatic and late seizures: risk factors. Eur J Neurol. 2013;20:1247–55. 23581284 10.1111/ene.12157

[19] Phan J Ramos M Soares T Parmar MS. Poststroke seizure and epilepsy: a review of incidence, risk factors, diagnosis, pathophysiology, and pharmacological therapies. Oxid Med Cell Longev. 2022;2022:7692215. 36338344 10.1155/2022/7692215 PMC9629926

[20] Misirli H Ozge A Somay G Erdoğan N Erkal H Erenoğlu NY. Seizure development after stroke. Int J Clin Pract. 2006;60:1536–41. 16669832 10.1111/j.1742-1241.2005.00782.x

[21] Graham NS Crichton S Koutroumanidis M Wolfe CD Rudd AG. Incidence and associations of poststroke epilepsy: the prospective South London Stroke Register. Stroke. 2013;44:605–11. 23370202 10.1161/STROKEAHA.111.000220

[22] Tanaka T Yamagami H Ihara M. Seizure outcomes and predictors of recurrent post-stroke seizure: a retrospective observational cohort study. PLoS One. 2015;10:e0136200. 26309124 10.1371/journal.pone.0136200 PMC4550357

[23] Ouerdiene A Messelmani M Derbali H. Post-stroke seizures: risk factors and management after ischemic stroke. Acta Neurol Belg. 2023;123:145–52. 34251613 10.1007/s13760-021-01742-x

